# The Impact of Selected Risk Factors on the Frequency of Cardiac Rehabilitation: Part I

**DOI:** 10.3390/jcm14238289

**Published:** 2025-11-21

**Authors:** Andrzej Kleinrok, Marlena Krawczyk-Suszek, Beata Zams

**Affiliations:** 1 Faculty of Health Sciences, Vincent Pol University, 20-816 Lublin, Poland; a.kleinrok@wp.pl; 2 Department of Physiotherapy, Medical College, University of Information Technology and Management, 35-225 Rzeszow, Poland; 3 Institute of Humanities and Medicine, Academy of Zamosc, 22-400 Zamosc, Poland; bzams@interia.pl

**Keywords:** cardiac rehabilitation, heart attack, hypertension, obesity, smoking

## Abstract

**Background/Objectives:** Cardiac rehabilitation (CR) is the most important element in the process of returning patients to everyday functioning. The aim of the study was to compare the frequency of use of various forms of CR in terms of the occurrence of various risk factors in patients after acute coronary syndrome (ACS). **Methods**: The study was conducted in a group of 1600 patients after ACS across 4 time points: 3, 6, 12 months, and 5 years. Patients were classified into four groups: (1) patients who do not participate in any rehabilitation programme after hospital treatment (0 + 0); (2) patients participating in a sanatorium programme (0 + S); (3) patients participating in an outpatient programme (0 + A); (4) patients participating in both forms (A + S). The study retrospectively analysed patients’ medical records for the following risk factors: smoking, hypertension, and obesity. The relationship between the four groups and the likelihood of undergoing a given form of rehabilitation was determined (Odds ratio [OR]; 95% confidence interval [CI]). Group matching in terms of gender and age was also applied using Propensity Score Matching (PSM). **Results:** Among those who did not undergo rehabilitation (0 + 0) 6 months after ACS, compared to the baseline groups, the most common were those who returned to smoking (OR = 3.42) and those with persistent obesity (OR = 3.69); after 5 years–individuals who reduced their obesity (OR = 4.86; after PSM: OR = 6.51). The highest chance of A + S rehabilitation was observed in the 3rd and 6th month in the group of people who quit smoking (OR = 7.60; OR = 6.04), after 6 months in the ‘reduction in uncontrolled hypertension’ group (OR = 11.44) and after 3 months in the ‘Reduction in obesity (after PSM)’ group (OR = 7.80). **Conclusions:** The most effective reduction in selected risk factors is reached by participation in sanatorium and ambulatory programmes (A + S).

## 1. Introduction

Rehabilitation is an integral part of patient treatment and, depending on the disease, the rehabilitation process has different priorities, with appropriate therapeutic methods taken into account. The implementation of the rehabilitation process also depends on the severity of the disease and the stage of its treatment, as well as on the patient’s abilities and acceptance of the methods used. Knowledge of the course and consequences of the disease allows the goal of rehabilitation to be determined. However, the effectiveness of rehabilitation depends not only on the patient’s commitment, but also on the professionalism of the staff. Knowledge of the mechanisms of action of the therapeutic methods used enables optimal selection of the components of the process and the order in which they are applied. In order to achieve a good rehabilitation effect, it is necessary to use several of the methods mentioned. Such a broad and interdisciplinary approach to the rehabilitation process is called comprehensive (holistic) rehabilitation [1,2,3,4,5]. One form of rehabilitation is cardiac rehabilitation (CR), which is widely recognised as a method of treating patients. It is part of secondary prevention of coronary heart disease and is divided into three main phases. In the early phase (phase 1), patient mobilisation is used during acute hospitalisation. Phase 2 includes rehabilitation services traditionally provided on an outpatient basis. The main goal of this phase is to change the patient’s health behaviours, modify risk factors (RF), and improve psychosocial well-being. Phase 3 involves the long-term maintenance of the lifestyle changes previously introduced by the patient [5,6].

Comprehensive cardiac rehabilitation (CCR) is a special type of rehabilitation that involves a multidisciplinary approach. CCR encompasses individual elements of comprehensive patient treatment, such as patient assessment, management and control of cardiovascular RF, promotion and awareness-raising of healthy physical activity, dietary advice, psychosocial and occupational support. Each of these elements of CCR is essential for public health—favourable rehabilitation outcomes, improved quality of life for patients, and optimal costs [5].

The most common indications for CCR are acute coronary syndrome (ACS), status after revascularisation procedures and status after surgical treatment of heart disease. CCR is also used in clinically evident coronary artery disease, the presence of strong RF, high cardiovascular risk, and heart failure [5].

In the holistic implementation of the CCR programme, identifying and eliminating risk factors (RFs) is extremely important. The five main RFs (hypertension, dyslipidaemia, overweight and obesity, diabetes and smoking) are responsible for 50% of cardiovascular diseases worldwide [7]. Therefore, 50% of all cases of cardiovascular disease (CVD) can be prevented by effectively eliminating the main RFs [7]. However, CVD significantly reduces patients’ quality of life; hence, measures should be implemented in this area [8,9,10]. Effective treatment of hypertension in people aged 55–60 adds the most years without CVD, and quitting smoking adds the most years of life [7]. Combating risk factors is particularly important. An analysis of the 10-year risk of death or cardiovascular disease in a population of 1.5 million healthy people worldwide showed that the presence of risk factors (systolic blood pressure, non-HDL cholesterol, diabetes and smoking) accounts for 57.2% of cardiovascular disease cases in women and 52.6% in men, and for 22.2% and 19.2% of deaths, respectively [11].

For the patient, the main goals of rehabilitation are as follows: the removal of symptoms, the consequences of the disease and its causative factors, as well as the restoration of the patient’s full or maximum possible physical, mental, social and professional fitness and enabling a return to independence through the restoration of fitness [12]. The basic condition for achieving positive rehabilitation results is cooperation between professional healthcare staff and the patient. Cooperation with the patient becomes effective when the patient has knowledge and understanding, which ensures their motivation [1,12,13].

According to the guidelines of the European Society of Cardiology (ESC), CR has been classified as a class IA method in the treatment of, among others, patients with ACS. Forms of CR may include outpatient intervention, rehabilitation in a sanatorium setting, and, according to the latest guidelines, may also be conducted in the form of telerehabilitation, with satisfactory therapeutic results [14]. Given the ageing population, varying percentages of ACS patients in different countries [15], the economic burden on national healthcare systems, and, above all, the high risk of recurrence, secondary prevention strategies are an important pillar of public health and quality of life for patients [16].

The aim of the study was to compare the frequency of use of different forms of CR in terms of the occurrence of various risk factors in patients after acute coronary syndrome.

## 2. Materials and Methods

### 2.1. Organisation of the Study

The retrospective analysis was conducted by medical records of 1600 patients admitted to the Cardiology Department of Provincial Hospital Pope John Paul II in Zamosc in 2017–2022. The authorities of the hospital and the University of Management and Administration gave consent to carry out the study. The study also received the positive opinion of the Commission on Ethics of Scientific Research of the University of Information Technology and Management in Rzeszow (No.2/2022). The reason for the admission was ACS diagnosed on the basis of chest pain, ECG teletransmission from ambulance and cardiological teleconsultation. The decision to admit a patient to the hospital, bypassing the nearest hospital, was made by the chief cardiologist on duty. The described system covered a population of 400,000 inhabitants.

### 2.2. Patients

The study included 1600 patients with ACS diagnosed on the basis of chest pain, ECG teletransmission from an ambulance, and cardiological teleconsultation. The majority of patients included in the study were men (69.7% of the total), with an average age of 58 ± 10 years. Most of the respondents lived in cities (54.0% of the total). Before ACS, most patients were in the working population (46.3%); after the disease, most retired (46.3%). Among the risk factors recorded, the majority of respondents had hypertension (56.3% of the total). In addition, smoking was recorded in 32.0% of the group, and the average BMI was 27.0 ± 3.8 kg/m^2^, which indicates overweight. Detailed data are presented in Table 1.

### 2.3. Hospital Treatment

All patients underwent coronary angiography and revascularisation (angioplasty or surgical coronary bypass). The procedure was performed immediately in the catheterisation lab within 12 h of the beginning of chest pain. After the procedure, patients were hospitalised in the Coronary Care Unit (CCU) for 24–48 h. In addition to the nursing and medical supervision, patients have been undergoing passive physiotherapy from the first day, to prevent the negative effects of immobilisation and self-service learning. Medically stable patients were transferred to the Cardiology Department or directly to the Cardiac Rehabilitation Department, where they stay for 5–7 days. During this period, the passive and active physical exercises were realised in 60 min sessions under the supervision of the physio-therapist. The size of loads corresponeded to the patients’ performance and capabilities. The sessions were carried out twice a day, 7 days a week, individually and in groups. Patients received oral and written information about the disease, its symptoms, treatment, and appropriate responses to symptom onset, as well as physical activity and diet. This information was also provided to the patient`s family members.

### 2.4. The Course of Treatment After Discharging from the Hospital

After discharge from the hospital, patients visited the cardiological outpatient department. During the visit, the health condition was assessed, and after checking for contraindications, the participation in CR programme at the cardiological sanatorium and/or at the outpatient (ambulatory) CR centre was proposed. After the patient’s acceptance decision, the patient was referred for a non-invasive examination of cardiac function and arrhythmias, and for a sanatorium stay and/or outpatient rehabilitation. The referral describes the current health status, medications and risk factors for coronary artery disease.

### 2.5. Rehabilitation Programme After Hospital Treatment

Stay in the sanatorium. The sanatorium programme included a stay of 1–3 weeks. Interval physical exercises were adapted to the results of the exercise test. Health education was conducted with particular emphasis on risk factors and their reduction. Dietary advice was given, as well as gymnastic exercises were performed. Ambulatory programme. The programme included 26 two-hour sessions held over 12 weeks. Interval: 30–45 min of physical exercise sessions were gradually increased by adapting to pulse limit. General gymnastics in both programmes included: warm-up, conditioning, strength and endurance exercises, followed by calming and relaxation exercises, supplemented with breathing exercises. Health education and dietary counselling for patients and their families were conducted. The consultations with a psychologist were conducted at the patient’s request or for medical necessity. A resuscitation course for patients’ families was conducted.

### 2.6. Analysed Data

Patients’ data were placed in records containing the date and course of hospital treatment, as well as their participation in the post-hospital CR programme. Sociodemographic and medical data were obtained from hospital and outpatient documentation. Medical data included the occurrence of hypertension, diabetes, BMI, smoking, dyslipidaemia, and everyday physical activity (Table 1). The obtained data were placed in an Excel database. Depending on the participation in various patient rehabilitation programmes patients population was divided into 4 groups: group 1—patient who do not participate in any rehabilitation programme after hospital treatment (0 + 0), group 2—patients participating in sanatorium programme (0 + S), group 3—patients participating in outpatient (ambulatory) programme (0 + A), group 4—patients participating in both programmes: ambulatory and sanatorium (A + S). Data on the frequency of use of individual forms of rehabilitation, presented in Table 2, were calculated relative to the number of respondents in each group. In the case of the variables ‘Return to smoking addiction’ and ‘Reduction in obesity (BMI ≥ 30)’, the Propensity Score Matching method was used in relation to gender and age. The data analysis included an assessment of the dependence of the frequency of use of a given form of rehabilitation between the four groups analysed.

### 2.7. Statistical Analysis

Quantitative data were presented as numbers and percentages, and quantitative data were presented using means and standard deviations (M ± SD). The analysis of linear relationships between variables was performed using the Chi-square Pearson test. The probability of occurrence of a factor was estimated by determining the odds ratio (OR) and 95% confidence interval (±95% Cl) based on the frequency of occurrence of the factor compared to the initial group.

In order to reduce errors arising from intergroup differences in studies and to correctly estimate the impact of the intervention, the Propensity Score Matching (PSM) method was used. In this method, non-experimental data allow for the estimation of the effect of the intervention by matching individuals who received the intervention CR to a group of patients who did not receive the intervention.

Statistical evaluation was performed using Statistica, version 13.3 (StatSoft Poland, Kraków, Poland).

## 3. Results

An analysis was conducted comparing a group of patients without rehabilitation with groups of patients receiving various forms of rehabilitation while accounting for risk factors. The frequency of individual rehabilitation forms was reported relative to the total number of respondents and the number of respondents in a given group. The aim of this analysis was to identify risk factors influencing participation in the CR programme. The verification included four consecutive time intervals, followed by a re-verification. In the smoking group, significant differences between groups were observed at each consecutive time interval (*p* < 0.05). In the first analysed period (3 months), ambulatory rehabilitation (A + 0; 34.0%) and sanatorium rehabilitation (0 + S; 33.2%) were used significantly more often among ACS patients. During this period, 28.6% of patients did not undergo any form of rehabilitation (0 + 0). In subsequent time intervals after 6 months, 12 months, and 5 years, 0 + S rehabilitation was consistently used significantly more often among patients, and both forms of rehabilitation—outpatient and sanatorium (A + S)—were used least often. The same trend was observed in the group of patients who returned to smoking (after matching the group using the PSM method), where 0 + S rehabilitation was used significantly more often, or no rehabilitation was used. In the group of people who quit smoking, the most common form of rehabilitation within 6 and 12 months was A + S, while after 3 months and after 5 years it was A + 0 (37.0% and 44.9%, respectively). Among patients with uncontrolled hypertension after 3 months, the most frequently used rehabilitation was A + 0 (38.4%) and 0 + S (24.1%). After 6 months, 35.4% of patients most often did not attend rehabilitation at all or used A + 0 (29.9%). After 12 months, 30.8% did not use rehabilitation, and 39.4% used 0 + S. After 5 years, most often patients participated in sanatorium rehabilitation (0 + S; 45.8%) or ambulatory rehabilitation (A + 0; 25.3%). In the group of patients who reduced uncontrolled hypertension at each time interval, the combined form of A + S or A + 0 rehabilitation was used significantly more often (*p* < 0.0001). Of the total number of patients studied, 20.1% had a BMI ≥ 30. In this group, two extreme patterns were most commonly observed—either the use of both forms of rehabilitation (A + S) or neither (0 + 0), with the exception of the period after 5 years. In contrast, among patients with persistent overweight, regardless of the time after ACS, significantly more often, both forms of rehabilitation were observed. In the group of patients who reduced obesity at each time interval, both forms of rehabilitation (A + S) were most commonly used, although after 12 months, the frequency of their use decreased. In contrast, after 12 months, A + 0 rehabilitation was used more frequently (30.3%). In the group after matching (PSM), A + S was more frequently used after 3, 6 months, and after 5 years, while after 12 months (47.4%), A + 0 was reported more frequently. Detailed data are presented in Table 2.

The impact of the analysed risk factors on participation in various forms of rehabilitation was assessed. People who returned to smoking addiction 3 months after ACS were twice as likely not to undergo rehabilitation compared to the group of smokers (OR = 1.96), and almost 3.5 times more likely after 6 months (OR = 3.42). After a period of 5 years, the rates of rehabilitation use (OR = 1.05) were similar in both groups. Those who returned to smoking were almost 1.5 times more likely to use sanatorium rehabilitation 3 months after ACS (OR = 1.55) compared to smokers. No significant differences were noted in other forms of rehabilitation. On the other hand, people who quit smoking were more than 7.5 times (OR = 7.60) more likely to use A + S rehabilitation after 3 months, 6 times (OR = 6.04) more likely after 6 months, and almost 3 times (OR = 2.75) more likely after 12 months. Patients with uncontrolled hypertension were about 3 times more likely after 6 (OR = 3.42) and 12 (OR = 2.75) months not to use any form of rehabilitation compared to other people with hypertension. In the group of patients with reduced hypertension, after 6 months, the use of both forms of rehabilitation (A + S) was more than 11 times more frequent compared to the 0 + 0 group (OR = 11.44) after 6 months, 7 times more often (OR = 7.40) after 12 months, and almost 5 times more often (OR = 4.91) after 5 years. Patients who remained overweight after 3 (OR = 2.08) and 12 (OR = 2.40) months were twice as likely not to use rehabilitation compared to the obese group, and after 6 months, almost four times more likely (OR = 3.69). A significantly higher likelihood of using rehabilitation in this group was observed 5 years after ACS. Patients with persistent overweight were more than five times (OR = 5.36) more likely to use 0 + S and 3 times (OR = 3.15) more likely to use A + S than the group of patients not using rehabilitation (0 + 0). People who reduced their obesity after 5 years were more than five times more likely not to use any form of rehabilitation (OR = 4.86) compared to people with obesity. In this group, A + S rehabilitation was used three times more often after 3 months (OR = 3.10), five times more often after 6 months (OR = 5.22) and almost three times more often after 12 months (OR = 2.77). In the group after PSM, the lack of rehabilitation 5 years after ACS was recorded 6 times more often compared to the remaining group of obese patients (OR = 6.51). In the group of patients after obesity reduction, 0 + S rehabilitation (OR = 2.04) was performed twice as often 3 months after the onset of the disease compared to the 0 + 0 group. A similar OR value was observed in the group of patients who underwent A + 0 rehabilitation (OR = 2.0) after 12 months. Patients after obesity reduction underwent A + S rehabilitation almost 8 times more often after 3 months (OR = 7.80), 4 times more often after 6 months (OR = 4.44) and 12 months (OR = 4.24). Detailed data are presented in Table 3.

**Table 2:** Risk factors in subsequent stages of assessment (3, 6, 12 months, and 5 years) in various forms of rehabilitation compared to the group without post-hospital rehabilitation.

Variables [n (%)]	Total N = 1600	A + 0 N = 412	0 + S N = 415	A + S N = 343	0 + 0 N = 430	*p* *
Smoking						
**Initially**	512 (32.0) **	140 (34.0)	138 (33.2)	111 (32.4)	123 (28.6)	0.5600
**After 3 months**	334 (65.2) ***	93 (27.8)	120 (35.9)	36 (10.8)	85 (25.4)	<0.0001
**After 6 months**	250 (48.8) ***	59 (23.6)	83 (33.2)	33 (13.2)	75 (30.0)	<0.0001
**After 12 months**	354 (69.1) ***	94 (26.6)	112 (31.6)	57 (16.1)	91 (25.7)	0.0031
**After 5 years**	232 (45.3) ***	62 (26.7)	80 (34.5)	41 (17.7)	49 (21.1)	0.0082
**Return to smoking addiction [after Propensity Score Matching (based on gender + age)]**						
**After 3 months**	325 (63.5) ***	92 (28.3)	118 (36.3)	30 (9.2)	85 (26.2)	<0.0001
**After 6 months**	240 (46.9) ***	58 (24.2)	82 (34.2)	27 (11.3)	74 (30.8)	<0.0001
**After 12 months**	340 (66.4) ***	90 (26.5)	111 (32.6)	51 (15.0)	88 (25.9)	<0.0001
**After 5 years**	219 (42.8) ***	55 (25.1)	78 (35.6)	37 (16.9)	49 (22.4)	0.0005
**Giving up smoking**						
**After 3 months**	81 (15.8) ***	30 (37.0)	11 (13.6)	32 (39.5)	18 (22.2)	<0.0001
**After 6 months**	146 (28.5) ***	61 (41.8)	42 (28.8)	31 (21.2)	12 (8.2)	0.0008
**After 12 months**	80 (15.6) ***	39 (48.8)	15 (18.8)	19 (23.8)	7 (8.8)	0.0003
**After 5 years**	156 (30.5) ***	70 (44.9)	47 (30.1)	25 (16.0)	14 (9.0)	0.0709
**The occurrence of uncontrolled hypertension**						
	Total N = 991	A + 0 N = 273	0 + S N = 266	A + S N = 343	0 + 0 N = 430	*p* *
**Initially**	505 (51.0) **	174 (34.5)	206 (40.8)	91 (18.0)	34 (6.7)	<0.0001
**After 3 months**	411 (81.4) ***	158 (38.4)	99 (24.1)	65 (15.8)	89 (21.7)	<0.0001
**After 6 months**	268 (53.1) ***	80 (29.9)	73 (27.2)	20 (7.5)	95 (35.4)	<0.0001
**After 12 months**	419 (83.0) ***	84 (20,0)	165 (39.4)	41 (9.8)	129 (30.8)	<0.0001
**After 5 years**	336 (66.5) ***	85 (25.3)	154 (45.8)	29 (8.6)	68 (20.2)	<0.0001
**Reduction in uncontrolled hypertension (systolic ≥140 or diastolic ≥90 mmHg) in patients with HA**						
**After 3 months**	401 (40.5) **	115 (28.7)	55 (13.7)	143 (35.7)	88 (21.9)	<0.0001
**After 6 months**	539 (54.4) **	193 (35.8)	80 (14.8)	188 (34.9)	78 (14.5)	<0.0001
**After 12 months**	489 (49.3) **	169 (34.6)	92 (18.8)	160 (32.7)	68 (13.9)	<0.0001
**After 5 years**	346 (34.9) **	118 (34.1)	98 (28.3)	88 (25.4)	42 (12.1)	<0.0001
**Obesity (BMI ≥ 30)**						
	Total N = 1600	A + 0 N = 412	0 + S N = 365	A + S N = 343	0 + 0 N = 234	*p* *
**Initially**	321 (20.1) ***	60 (18.7)	57 (17.8)	105 (32.7)	99 (30.8)	<0.0001
**After 3 months**	254 (79.1) ***	61 (24.0)	40 (15.7)	88 (34.6)	65 (25.6)	<0.0001
**After 6 months**	205 (63.9) ***	45 (22.0)	37 (18.0)	71 (34.6)	52 (25.4)	<0.0001
**After 12 months**	222 (69.2) ***	47 (21.2)	37 (16.7)	84 (37.8)	54 (24.3)	<0.0001
**After 5 years**	155 (48.3) ***	38 (24.5)	42 (27.1)	56 (36.1)	19 (12.3)	0.0058
**Persistent obesity (BMI ≥ 30)**						
	Total N = 321	A + 0 N = 60	0 + S N = 57	A + S N = 105	0 + 0 N = 99	*p* *
**After 3 months**	241 (75.1) ***	55 (22.8)	40 (16.6)	84 (34.9)	62 (25.7)	0.0409
**After 6 months**	199 (62.0) ***	44 (22.1)	36 (18.1)	69 (34.7)	50 (25.1)	0.0003
**After 12 months**	191 (59.5) ***	37 (19.4)	35 (18.3)	68 (35.6)	51 (26.7)	0.0037
**After 5 years**	118 (36.8) ***	26 (22.0)	38 (32.2)	43 (36.4)	11 (9.3)	<0.0001
**Reduction of obesity (BMI ≥ 30)**						
**After 3 months**	35 (10.9) ***	5 (14.3)	4 (11.4)	21 (60.0)	5 (14.3)	0.0409
**After 6 months**	60 (18.7) ***	16 (26.7)	3 (5.0)	36 (60.0)	6 (10.0)	0.0003
**After 12 months**	76 (23.7) ***	23 (30.3)	6 (7.9)	37 (48.7)	10 (13.2)	<0.0001
**After 5 years**	150 (46.7) ***	32 (21.3)	6 (4.0)	62 (41.3)	50 (33.3)	<0.0001
**Reduction of obesity (BMI ≥ 30) [after Propensity Score Matching (based on gender + age)]**						
**After 3 months**	19 (5.9) ***	2 (10.5)	4 (21.2)	12 (63.2)	1 (5.3)	0.0202
**After 6 months**	30 (9.3) ***	10 (33.3)	2 (6.7)	16 (53.3)	2 (6.7)	0.0410
**After 12 months**	38 (11.8) ***	18 (47.4)	4 (10.5)	14 (36.8)	2 (5.3)	0.0124
**After 5 years**	73 (22.7) ***	23 (31.5)	3 (4.1)	27 (37.0)	20 (27.4)	<0.0001

A + 0—ambulatory rehabilitation; 0 + S—sanatorium programme; A + S—sanatorium and ambulatory; 0 + 0—without rehabilitation; *p* *—*p* value X^2^ Pearson test analysis of the relationship between 4 groups (forms of rehabilitation); [n (%)]—percentage of the number of people in a given group [3, 6, 12 months, 5 years]; **—percentage of the total number of people; ***—percentage of the initial number of people.

**Table 3:** Intensity [Odds Ratio] of risk factors at subsequent stages of assessment (3, 6, 12 months and 5 years) across various forms of rehabilitation compared to the group without post-hospital rehabilitation.

Variables	0 + 0 ^1^ [OR (±95 Cl)]	0 + S ^2^ [OR (±95 Cl)]	A + 0 ^2^ [OR (±95 Cl)]	A + S ^2^ [OR (±95 Cl)]
Return to smoking addiction [after Propensity Score Matching (based on gender + age)]				
**After 3 months**	1.96 (1.20–3.20)	1.55 (1.20–4.83)	0.84 (0.33–1.03)	0.11 (0.06–0.21)
**After 6 months**	3.42 (2.12–5.53)	0.79 (0.59–1.05)	0.64 (0.15–0.70)	0.12 (0.06–0.26)
**After 12 months**	2.08 (1.25–3.48)	1.15 (0.82–1.60)	0.77 (0.63–0.93)	0.21 (0.12–0.39)
**After 5 years**	1.05 (0.68–1.60)	1.31 (1.01–1.69)	0.92 (0.77–1.09)	0.60 (0.34–1.04)
**Giving up smoking**				
**After 3 months**	0.96 (0.40–2.28)	0.54 (0.32–0.91)	0.96 (0.70–1.31)	7.60 (2.50–23.07)
**After 6 months**	0.42 (0.16–1.07)	1.22 (0.74–2.01)	1.38 (0.99–1.91)	6.04 (1.90–19.15)
**After 12 months**	0.75 (0.29–1.96)	0.74 (0.43–1.27)	1.22 (0.87–1.70)	2.75 (0.90–8.41)
**After 5 years**	0.73 (0.32–1.67)	0.96 (0.62–1.50)	1.23 (0.17–1.23)	1.59 (0.58–4.40)
**The occurrence of uncontrolled hypertension**				
**After 3 months**	1.02 (0.86–1.20)	1.33 (1.07–1.66)	1.10 (0.87–1.26)	0.45 (0.30–0.68)
**After 6 months**	3.42 (2.29–4.59)	0.86 (0.69–1.08)	0.70 (0.61–0.80)	0.09 (0.05–0.15)
**After 12 months**	2.75 (1.98–3.83)	0.97 (0.80–1.18)	0.64 (0.56–0.73)	0.13 (0.08–0.21)
**After 5 years**	1.83 (1.21–2.79)	0.98 (0.78–1.25)	0.76 (0.65–0.89)	0.20 (0.11–0.36)
**Reduction of uncontrolled hypertension (systolic ≥140 or diastolic ≥90 mmHg) in patients with HA**				
**After 3 months**	0.98 (0.82–1.24)	0.75 (0.60–0.94)	0.90 (0.79–1.02)	2.22 (1.47–3.38)
**After 6 months**	0.31 (0.22–0.44)	1.16 (0.93–1.44)	1.43 (1.25–1.64)	11.44 (6.59–19.90)
**After 12 months**	0.36 (0.26–0.51)	1.03 (0.85–1.25)	1.56 (1.37–1.78)	7.40 (4.70–11.45)
**After 5 years**	0.54 (0.36–0.83)	1.01 (0.80–1.28)	1.31 (1.12–1.54)	4.91 (2.77–8.71)
**Persistent obesity (BMI ≥ 30)**				
**After 3 months**	2.08 (0.78–5.61)	0.90 (0.45–1.80)	0.96 (0.62–1.48)	0.32 (0.11–0.91)
**After 6 months**	3.69 (1.39–9.78)	1.09 (0.51–2.33)	0.65 (0.45–0.94)	0.19 (0.07–0.53)
**After 12 months**	2.40 (1.10–5.05)	1.07 (0.61–1.87)	0.68 (0.51–0.90)	0.36 (0.16–0.80)
**After 5 years**	0.21 (0.10–1.42)	5.36 (3.10–9.27)	1.54 (1.17–2.05)	3.15 (1.46–6.78)
**Reduction of obesity (BMI ≥ 30)**				
**After 3 months**	0.48 (0.18–1.30)	1.11 (0.56–2.23)	1.04 (0.67–1.60)	3.10 (1.10–8.74)
**After 6 months**	0.27 (0.10–0.72)	0.91 (0.43–1.95)	1.54 (1.07–1.21)	5.22 (1.90–14.35)
**After 12 months**	0.42 (0.20–0.87)	0.93 (0.56–1.63)	1.47 (1.10–1.96)	2.77 (1.25–6.13)
**After 5 years**	4.86 (2.39–9.90)	0.19 (0.11–0.32)	0.65 (0.49–0.86)	0.32 (0.15–0.68)
**Reduction of obesity (BMI ≥ 30) [after Propensity Score Matching (based on gender + age)]**				
**After 3 months**	0.23 (0.03–1.82)	2.04 (0.64–6.48)	1.07 (0.47–2.47)	7.80 (0.92–65.90)
**After 6 months**	0.34 (0.07–1.54)	0.91 (0.32–2.61)	1.43 (0.83–2.48)	4.44 (0.90–21.93)
**After 12 months**	0.21 (0.05–0.94)	1.44 (0.58–3.63)	2.00 (1.17–3.40)	4.24 (0.86–20.90)
**After 5 years**	6.51 (2.08–20.39)	0.16 (0.07–0.35)	0.62 (0.41–0.95)	0.22 (0.06–0.73)

A + 0—ambulatory rehabilitation; 0 + S—sanatorium programme; A + S—sanatorium and ambulatory; 0 + 0—without rehabilitation; OR—Odds Ratio; ±95 Cl—95% confidence interval; ^1^—relative to: the remaining group with uncontrolled hypertension [for the analysis of hypertension], the remaining group of initial smokers [for the analysis of smoking], the remaining group with obesity [for the analysis of obesity]; ^2^—relative to group 0 + 0.

## 4. Discussion

The benefits of CCR are widely recognised. They can be divided into physiological, anatomical, psychological, epidemiological and economic benefits [5,17,18]. CCR is based on physical training and modification or elimination of RF (smoking addiction, lipid disorders, body weight, blood pressure, etc.) [19]. Hence, the authors included smoking, hypertension, and obesity in their analysis as factors modifying participation in CR. The most important benefits of CCR include a 26% reduction in cardiovascular mortality, a 23% reduction in hospitalisation rates, an 18% reduction in recurrent heart attacks, as well as an improvement in quality of life and a reduction in treatment costs [19].

The CCR process can be carried out in a hospital, an outpatient clinic, a sanatorium, or at the patient’s home, depending on the patient’s goals, capabilities, and preferences. The CR process can be divided into three [13,18] or four stages [4]. Both divisions are similar in terms of their goals and methods of implementation. The entire CCR process can also be divided into hospital and post-hospital parts. The post-hospital part of CCR can be divided into two periods: an active, organised period carried out in a sanatorium or outpatient CCR centre, and a passive, unorganised period in which the patient follows the principles of a healthy lifestyle individually or in groups.

The first stage of CCR is carried out during the patient’s stay in the hospital. It applies to patients in the acute phase of the disease. It takes place in Coronary Care Units (CCUs), Post-Operative Wards, Cardiac Rehabilitation Wards or specialist hospital wards. The implementation of the CR programme in the acute phase of the disease results in a reduction in hospitalisation time, mortality, and the need for repeat revascularisation procedures [4]. The aim of the first stage is to help the patient achieve independence in everyday life as quickly as possible, counteract the effects of immobilisation and provide basic knowledge about the disease, its consequences and RF. In recent decades, there has been a systematic reduction in the length of hospitalisation for patients with ACS. Patients treated invasively with coronary angioplasty are discharged from the hospital within 4–7 days, provided there are no complications. For these reasons, the CR hospital programme for these patients is more informative than therapeutic in nature. The prerequisite for achieving the set goals is the implementation of CCR [4,5].

The second stage is carried out at home, in a sanatorium and/or in an outpatient centre. The patient’s place of residence is a common location for the second stage. Such a location for the rehabilitation process is usually limited in scope, without supervision or the possibility of correction, and is not very effective. A more effective CCR option is to participate in a CCR programme at a sanatorium, an outpatient centre, or both, due to the possibility of active interaction and cooperation between the patient and medical staff and the provision of appropriate motivation and healthy habits. An important goal of the second stage is to implement a healthy lifestyle through education on combating RF, mainly smoking, blood pressure control, weight reduction, reduction of emotional tension, and reduction of the sense of threat, which leads to an improvement in quality of life [3,17,18,20].

CCR programmes in Europe usually last between 2 and 24 weeks, while in the USA, they last between 6 and 36 weeks [4]. A comparison of the effectiveness of CCR in reducing blood pressure showed that longer programmes were more effective [21]. In the current study, we did not analyse the duration of rehabilitation, but the combination of A + S rehabilitation, which resulted in a longer duration of rehabilitation attendance, and in the group of patients with reduced uncontrolled hypertension, the chances of undergoing both forms of rehabilitation were 11 times higher (OR = 11.44) in this group compared to the initial group. This confirms that the reduction in hypertension was the result of several forms of rehabilitation, which were associated with a longer duration of rehabilitation. However, duration strengthens the patient’s motivation.

The implementation of stage II in an organised form results in an improvement in prognosis, as assessed by hard points. In a group of patients after myocardial infarction participating in an outpatient rehabilitation programme, a reduction in the incidence of MACE (Major Adverse Cardiac Events) was demonstrated, along with reductions in lipid concentrations and BMI and an increase in left ventricular ejection fraction (EF) [22]. A meta-analysis of 31 studies involving nearly 230,000 patients after ACS showed a beneficial effect of CCR on all-cause mortality (HR in the prospective analysis was 0.37 and OR 0.20, and in the retrospective analysis, HR was 0.64) [23]. In turn, a meta-analysis of 85 randomised studies involving nearly 23,000 patients after myocardial infarction showed a 26% reduction in annual cardiovascular mortality, a 23% reduction in hospitalisations, an 18% reduction in recurrent myocardial infarctions [4,19] and an improvement in quality of life [24].

International research conducted as part of the INTERASPIRE project shows that CR is recommended to an average of 34.4% of patients, while in Poland, this percentage was 69.6%. Only 19.6% of the patients surveyed participated in ≥50% of the sessions, which was likely strongly associated with the final outcomes of rehabilitation. Furthermore, this percentage was associated with a lower incidence of persistent smoking and lack of physical activity, better control of blood pressure and LDL cholesterol levels, and more frequent use of cardioprotective drugs [16].

In the current study, we compared four different CR regimens, and thus different forms such as outpatient and sanatorium rehabilitation. The research indicates a greater likelihood of conducting A + S rehabilitation in individual groups of patients with risk factors than in the initial groups, or of not receiving rehabilitation, compared to other people who do not attend rehabilitation (0 + 0). However, in the case of A + 0 and 0 + S rehabilitation, no significant differences were observed, except among individuals with persistent obesity. In this group, there was a 5-fold higher likelihood (OR = 5.36) of participating in A + 0 rehabilitation compared with the group with obesity.

According to research, multi-component rehabilitation programmes produce better therapeutic effects than single forms of treatment [25,26], which is why A + S rehabilitation was included among the rehabilitation programmes in our studies. We showed a significant frequency of use of this combined rehabilitation in a group of patients quitting smoking, where the chance of using this rehabilitation regimen was more than seven times higher compared to those not using rehabilitation (0 + 0) after 3 months (OR = 7.60) from ACS and six times higher after 6 months (OR = 6.04). The longer the time since ACS, the lower the chance of using rehabilitation. This trend was observed in each of the analysed risk factor groups, except for persistent obesity (BMI ≥ 30). In this group, 5 years after ACS, the chance of participating in A + S rehabilitation was more than 3 times higher (OR = 3.15) compared to the remaining group of obese individuals.

CR is a class 1 recommendation according to available and applicable guidelines, but according to available data, it is rarely recommended to patients in European countries, as shown in EUROASPIRE studies [27,28]. Therefore, it is important for national cardiology societies to take action to emphasise the importance of CR in the comprehensive treatment and rehabilitation of patients after ACS.

The current studies form the basis for further extensive analyses assessing the severity of risk factors in a group of patients with cardiovascular diseases and their impact on patients’ quality of life.

### Limitation

The first part of the analysis allowed for the assessment of the feasibility of conducting specific forms of rehabilitation for a group of patients, taking into account various risk factors. It did not include a detailed analysis of ACS risk factors for specific types of CR. Their inclusion in the analyses will allow a detailed assessment of how the type of rehabilitation used affected the level of individual risk factor parameters. Therefore, in the next parts of the analysis, the authors will present a detailed analysis of systolic blood pressure levels, an analysis of average BMI values, an analysis of MET (Metabolic Equivalent of Task)—average energy expenditure during everyday activities by patients participating in this study and differences between females and males.

## 5. Conclusions

The conclusion of this study is that cardiac rehabilitation has a positive effect on some risk factors in short- and long-term observation. The therapeutic effect is influenced by the type of rehabilitation used, indicating that an outpatient cardiac rehabilitation programme appears to be more effective than one delivered in a sanatorium. The factors that contributed to an increased likelihood of participation in A + S rehabilitation, especially in the period 3 and 6 months after ACS, were: smoking cessation, reduction of uncontrolled hypertension, and reduction of obesity (BMI ≥ 30). However, the best results of CR are reached by participation in both sanatorium and ambulatory programmes.

## Figures and Tables

**Table 1:** General characteristics of the group.

Variable [n (%)]	Total N = 1600 (100.0)	A + 0 N = 412 (25.8)	0 + S N = 415 (25.9)	A + S N = 343 (21.4)	0 + 0 N = 430 (26.9)	*p*
**Gender (male)**	1116 (69.7)	252 (61.2)	265 (63.9)	265 (77.3)	334 (77.4)	<0.0001
**Age (years)**	58.0 ± 10.0	57.5 ± 10.2	58.2 ± 11.0	56.8 ± 9.2	59.2 ± 9.3	<0.0001
**Place of residence (city)**	864 (54.0)	223 (54.1)	201 (48.4)	229 (66.8)	211 (49.1)	<0.0001
**Marital status** (married)	1250 (78.1)	281 (71.9)	313 (75.4)	259 (86.0)	361 (84.7)	<0.0001
**Education** (secondary or higher education)	718 (44.9)	169 (41.0)	192 (46.3)	169 (49.3)	188 (44.8)	0.1432
**Employment status at the time of admission:** — Employed — Unemployed — Retired — Disabled — Unknown	741 (46.3) 43 (2.7) 546 (34.1) 194 (12.1) 76 (4.8)	168 (40.8) 18 (4.4) 125 (30.3) 61 (14.8) 40 (9.7)	205 (49.4) 11 (2.7) 152 (36.6) 17 (4.1) 30 (7.2)	158 (46.1) 10 (2.9) 100 (29.1) 75 (21.9) 0 (0.0)	210 (48.9) 4 (0.9) 163 (39.3) 41 (9.5) 6 (1.4)	<0.0001
**Current employment status:** — Employed — Unemployed — Retired — Disabled — Unknown	496 (31.0) 36 (2.3) 741 (46.3) 261 (16.3) 66 (4.1)	137 (33.3) 15 (3.6) 152 (36.9) 79 (19.2) 29 (7.0)	145 (35.0) 8 (1.9) 207 (49.9) 42 (10.1) 13 (3.1)	115 (33.5) 11 (3.2) 121 (35.3) 90 (26.2) 6 (1.8)	99 (23.0) 2 (0.5) 261 (60.7) 50 (11.6) 18 (4.2)	<0.0001
**The illness was the reason for taking retirement**	86 (5.4)	25 (6.1)	29 (7.0)	6 (1.7)	26 (6.1)	<0.0001
**Medical data. risk factors and comorbidities (upon discharge from the hospital)** [M ± SD]						
**Physical activity** [min/week]	342.7 ± 437.8	336.2 ± 440.3	102.5 ± 64.7	788.6 ± 535.4	169.0 ± 83.9	<0.0001
**BMI** [kg/m^2^]	27.0 ± 3.8	26.6 ± 3.6	26.2 ± 3.1	27.9 ± 4.1	27.4 ± 4.2	0.0158
**Diabetes mellitus** [n (%)]	332 (20.8)	77 (18.7)	132 (31.8)	51 (14.9)	72 (16.7)	<0.0001
**Hypertension** [n (%)]	901 (56.3)	238 (57.8)	228 (54.9)	199 (58.0)	236 (54.9)	0.1615
**Systolic blood pressure** [mmHg]	131 ± 18.1	131.1 ± 17.3	136.3 ± 17.2	124.8 ± 14.7	131.6 ± 26.3	<0.0001
**Diastolic blood pressure** [mmHg]	80.1 ± 24.6	81.6 ± 39.9	83.2 ± 9.3	75.4 ± 9.6	77.9 ± 11.8	<0.0001
**Smoking [n (%)]:** — **Yes** — **No** — **Former smoker**	512 (32.0) 938 (58.6) 122 (7.6)	140 (34.0) 240 (58.2) 32 (7.8)	138 (43.0) 268 (66.0) 0 (0.0)	111 (32.4) 143 (41.7) 89 (25.9)	123 (29.9) 287 (69.9) 1 (0.2)	<0.0001
**Left Ventricular Ejection Fraction (LVEF)** [%]	54.5 ± 9.5	53.3 ± 9.4	51.1 ± 7.9	59.4 ± 8.1	54.6 ± 12.2	<0.0001
**Hyperlipidemia** [n (%)]	680 (42.5)	185 (44.9)	265 (63.9)	45 (13.1)	185 (43.0)	<0.0001

A + 0—ambulatory rehabilitation; 0 + S—sanatorium programme; A + S—sanatorium and ambulatory; 0 + 0—without rehabilitation; *p*—*p* value X^2^ Pearson test analysis of the relationship between 4 groups (forms of rehabilitation).

## Data Availability

Data will be available upon request.

## Abbreviations

The following abbreviations are used in this manuscript:

ACS	Acute coronary syndrome
BMI	Body mass index
CR	Cardiac rehabilitation
CCR	Comprehensive cardiac rehabilitation
CCU	Coronary care unit
HA	Heart attack
HDL	High-density lipoprotein
LDL	Low-density lipoprotein
Non-HDL	Non-high-density lipoprotein
MET	Metabolic Equivalent of Task
OR	Odds ratio
PSM	The propensity score matching
RF	Risk factor
